# Patients With Stage III PLCNEC Advocated for Radiotherapy Combined With Chemotherapy While With Stage IV Focus on Individualized Management: Evidence From SEER

**DOI:** 10.1002/cnr2.70573

**Published:** 2026-06-03

**Authors:** Yuze Zhao, Guantong Liu, Haitao Li, Yu Li, Ting Lin, Zefeng Xie

**Affiliations:** ^1^ The First Affiliated Hospital of Shantou University Medical College Shantou Guangdong China; ^2^ Chenghai People Hospital Shantou Guangdong China

**Keywords:** chemoradiotherapy, nomogram, prognosis, pulmonary large cell neuroendocrine carcinoma, survival analysis

## Abstract

**Background:**

PLCNEC is a rare and highly aggressive lung cancer with a poor prognosis. Multimodal therapy, primarily chemotherapy‐based, is the standard for advanced PLCNEC, but the role of radiotherapy remains unclear.

**Methods:**

We analyzed 1086 PLCNEC patients receiving chemotherapy from the Surveillance, Epidemiology, and End Results (SEER) database between 2004 and 2015. Propensity score matching (PSM) was applied to adjust baseline confounding variables. Kaplan–Meier survival curves were constructed and compared to assess survival differences. Cox proportional hazards regression models were employed to evaluate prognostic factors. Predictive nomograms were developed and validated to predict individual survival probabilities and facilitate clinical decision‐making.

**Results:**

Chemotherapy combined with radiotherapy was associated with improved OS and CSS compared with chemotherapy alone in stage III–IV PLCNEC patients (*p* < 0.05). Sex, N stage, M stage, surgical treatment, and radiotherapy were identified as independent prognostic factors. Nomograms developed demonstrated strong predictive performance, with areas under the curve (AUCs) for OS of 0.720, 0.821, and 0.847 at 1, 3, and 5 years, respectively, and for CSS of 0.733, 0.800, and 0.810 at the corresponding time points. Subgroup analyses suggested that selected patients may derive differential benefit from chemoradiotherapy.

**Conclusion:**

Radiotherapy is associated with improved survival outcomes in stage III PLCNEC patients receiving chemotherapy, whereas its role in stage IV disease appears more context‐dependent and warrants individualized consideration. The proposed nomograms provide complementary prognostic information to support individualized clinical decision‐making.

## 1 Introduction

According to the latest GLOBOCAN estimates, lung cancer remains the most commonly diagnosed cancer and the leading cause of cancer‐related death worldwide [1]. In 2022, almost 2.5 million new cases were diagnosed globally, and more than 1.8 million deaths were attributed to the disease, representing about 12% of all new cancers and around 18%–19% of all cancer deaths [2]. Traditionally, lung cancer has been categorized into two primary histological subtypes: small cell lung cancer (SCLC) and non‐small cell lung cancer (NSCLC). Pulmonary neuroendocrine tumors, a heterogeneous subtype of lung cancer characterized by neuroendocrine morphology, immunohistochemistry revealing neuroendocrine differentiation, a high mitotic rate (> 10 mitosis·2 mm^−2^) and non‐small cell cytological features, account for approximately 3% of lung cancers [3, 4, 5]. Despite their relatively low incidence, high‐grade pulmonary neuroendocrine carcinomas contribute disproportionately to lung‐cancer–related mortality due to their aggressive biological behavior and tendency to present at advanced stages.

In the past, lung neuroendocrine tumors have been histologically classified into three main groups: typical carcinoid (TC), atypical carcinoid (AC) and SCLC. Subsequently, Large cell neuroendocrine carcinoma (LCNEC) was designated by the World Health Organization as a fourth category lung neuroendocrine tumor due to its distinctive clinical features, histology, prognosis, and survival [3]. Pulmonary large cell neuroendocrine carcinoma (PLCNEC) is highly aggressive, rare, and challenging to diagnose, and optimal treatment strategies have not yet been clearly established, resulting in a generally poor prognosis [6]. Notably, approximately 40%–70% of PLCNEC patients are diagnosed at stage III–IV, highlighting the urgent need for improved more effective therapeutic strategies in advanced disease [7]. Currently, surgery or surgery combined with platinum‐based adjuvant chemotherapy is recommended as the mainstay of treatment for patients with stage I–III early‐stage PLCNEC [8], whereas multimodality therapy with chemotherapy is the preferred approach for progressive and advanced stages [9, 10]. Although radiotherapy is one of the treatment options for patients who cannot undergo surgical resection, its therapeutic value in stage III–IV disease remains uncertain and inconsistently reported in the literature [11].

A nomogram is a tool commonly utilized to evaluate prognosis by incorporating multiple prognostic and clinical decision‐related variables to calculate individualized numerical probabilities of clinical events, with the objective of maximizing predictive accuracy [12, 13]. The fundamental principle of the nomogram is to predict prognosis by assigning prognostic weights to each of the predicted disease parameters and combining them [12]. Despite the proliferation of nomograms [13], there remains limited evidence evaluating the prognostic impact of radiotherapy specifically in stage III–IV PLCNEC patients receiving chemotherapy. Moreover, no predictive nomogram has been developed for this advanced‐stage population, leaving clinicians without practical tools for individualized prognostication. Consequently, the present study utilized the Surveillance, Epidemiology, and End Results (SEER) database to evaluate the association between radiotherapy and survival outcomes in stage III–IV PLCNEC patients treated with chemotherapy, and develop a prognostic nomogram to support individualized survival prediction in this clinically challenging population. By addressing these gaps, this study aims to provide evidence‐based support for treatment decision‐making and improve risk stratification for advanced PLCNEC.

## 2 Methods

### 2.1 Data Sources and Patients

The SEER database is a publicly available cancer database that provides a wealth of clinical information on cancer patients. The SEER study data includes patient incidence and demographic data related by age, sex, race, year of diagnosis, and geographic area (including SEER registries and counties) (https://seer.cancer.gov/). We have obtained the requisite licensing through the formal signature of the SEER Study Data Agreement, thereby becoming authorized to access and utilize the data from the public SEER database. The anonymization and de‐identification of the data ensure that the study did not require institutional review board review or informed consent, in accordance with institutional and national guidelines.

**FIGURE 1:**
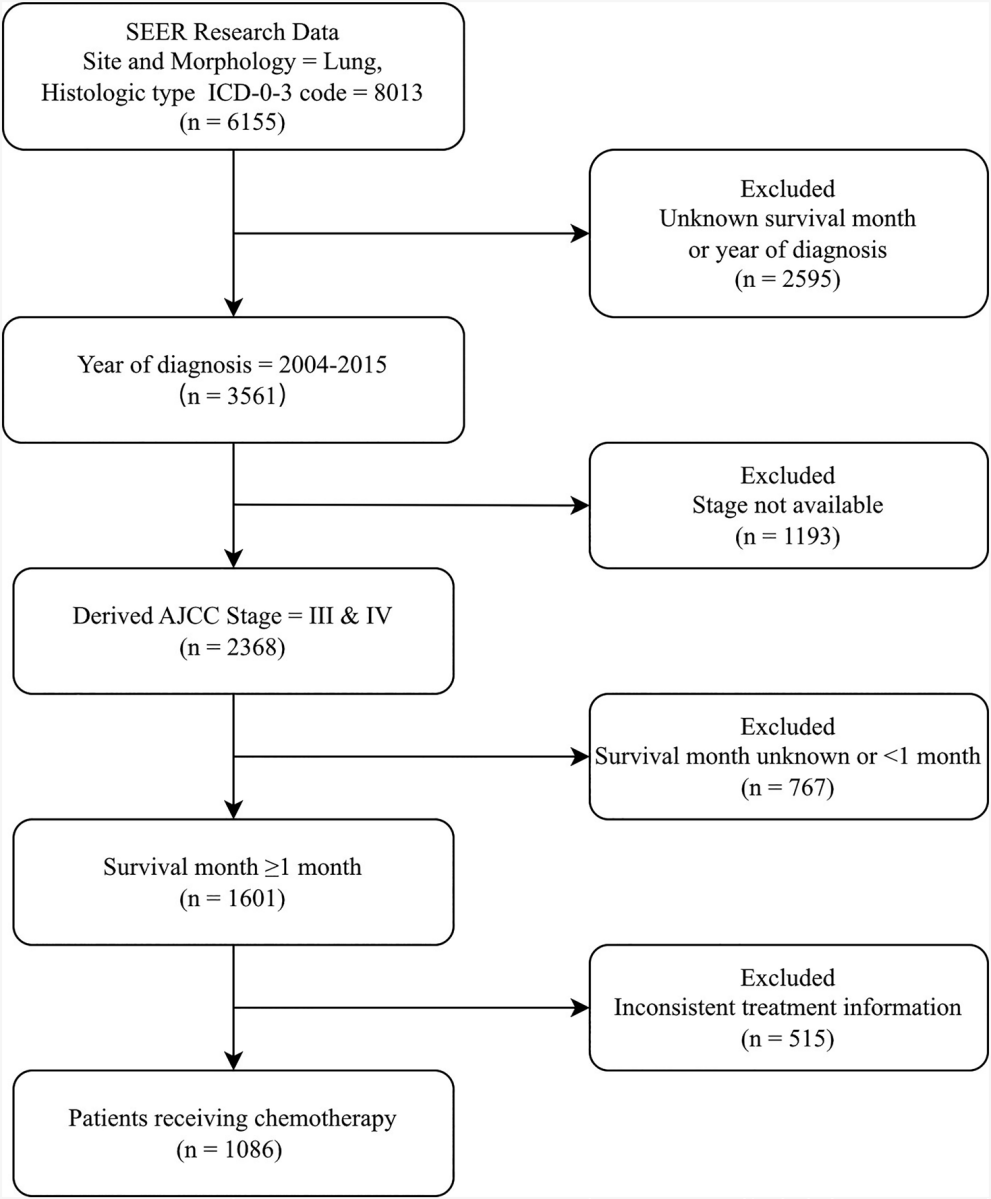
The flow chart for screening patients.

The patient population of this study was derived from the SEER*Stat v8.4.4 Incidence‐SEER Research Data, 17 Registries, Nov 2023 Sub (2000–2021) database. The inclusion criteria for the study were as follows: (1) Coding “Site and Morphology. CS Schema—AJCC 6th Edition” as “Lung.” (2) Coding “Site and Morphology. Histology Type ICD‐O‐3” as 8013. (3) Patients diagnosed between 2004 and 2015. The sixth edition of the AJCC clinical stage was used to determine the stage of the disease, with stage III to IV being the applicable stages. Prior chemotherapy was a prerequisite for inclusion in the study. The following criteria were used to exclude subjects from the study: (1) Patients with missing survival data. (2) Patients with survival time less than 1 month. (3) Patients with uncertain tumor size (Figure 1).

### 2.2 Variables

The following were variables of interest: Survival data: survival month, overall survival (OS), and cancer‐specific survival (CSS). OS is defined as the time interval from a cancer diagnosis to death or the most recent follow‐up visit. CSS is defined as the time interval from a cancer diagnosis to death due to cancer. The following variables were collected: age, sex, race, marital status, laterality, stage, T stage, N stage, M stage, tumor size (cm), primary site, grade, surgery, radiotherapy, survival time (month), survival status, and time since diagnosis. It is important to note that tumor staging was based on the sixth edition of the American National Joint Committee on Cancer's TNM staging (AJCC 6th Edition) (https://link.springer.com/book/10.1007/978‐1‐4757‐3656‐4) developed.

### 2.3 Statistical Analysis

**FIGURE 2:**
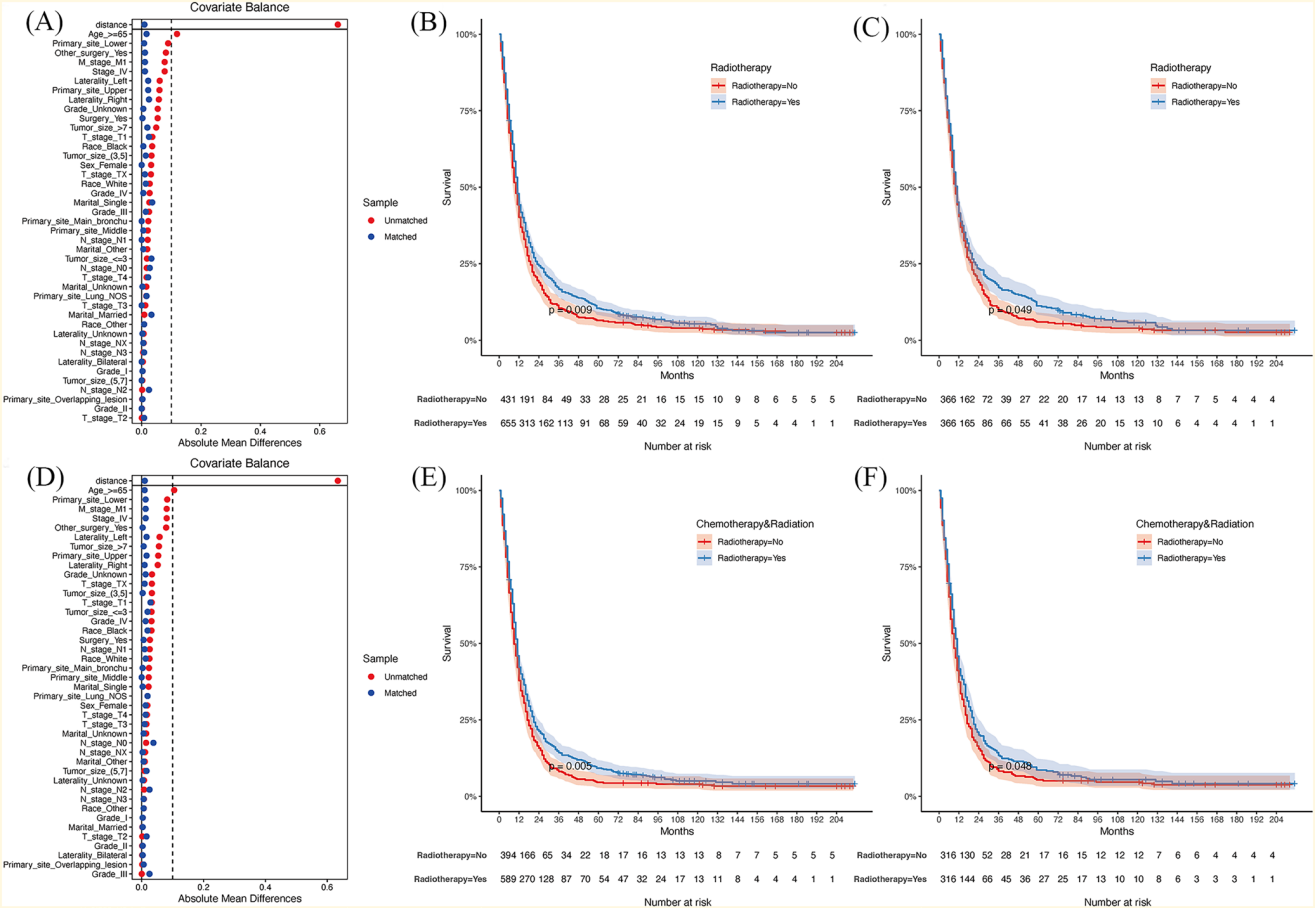
(A) Standardized mean differences before and after PSM (OS); (B) Comparison of OS before PSM; (C) Comparison of OS after PSM; (D) Standardized mean differences before and after PSM (CSS); (E) Comparison of CSS before PSM; (F) Comparison of CSS after PSM. Abbreviation: OS, overall survival; PSM, propensity score matching; CSS, cancer‐specific survival.

In this study, the chi‐square test was employed to compare baseline characteristics between groups. Propensity score matching (PSM) was employed to analyze patients who received radiotherapy versus those who did not, with the objective of reducing the effect of selection bias. The ratio of subjects who received radiotherapy to those who did not was maintained at 1:1, and the effect of covariates was balanced by the “nearest” method with a caliper value of 0.02. Standardized mean differences (SMD) were employed to assess the balance of data when the SMD was less than 0.1. When SMD < 0.1, the variable was considered to be well balanced between groups (Figure 2A,D). Survival curves plotted by the Kaplan–Meier (KM) method were used to compare the prognosis of the different treatments, and comparisons between curves were made using the log‐rank test.

The multicollinearity test was employed to ascertain the presence of high correlation between predictor variables. Multicollinearity can be diagnosed by the generalized variance inflation factor (GVIF), which is present if the GVIF is greater than 5 to 10 and if the adjusted GVIF is greater than two. The patient population was randomly allocated 7:3 into a training and validation set, with 70% of patients constituting the training cohort and 30% as the validation cohort. Independent prognostic factors were identified using univariate Cox regression analysis, and statistically significant variables were screened for inclusion in multifactorial Cox regression analysis. In this study, all independent prognostic factors from the results of the multivariate analysis were used to construct a nomogram model, and the predictive performance of the model was assessed by plotting its Receiver Operating Characteristic (ROC) Curves, Calibration Curves, and Decision Curve Analysis (DCA) curves. Categorical variables that were significant in univariate analyses were selected as subgroups, and the results of the subgroup analyses were presented using forest plots. Statistically significant results were defined as those with *p*‐values less than 0.05. All statistical analyses were performed using R software (version 4.4.1).

## 3 Results

### 3.1 Clinical Features

This study reviewed a total of 1086 patients, of whom 55.2% (*n* = 599) were male. Most patients exhibited unilateral laterality (98.4%, *n* = 1069), clinical stage IV (62.6%, *n* = 680), T4 stage (43.1%, *n* = 468), N2 stage (51.4%, *n* = 558), and M1 stage (62.6%, *n* = 680). Most patients did not receive surgical treatment (82.6%, *n* = 897) and did not undergo surgery other than the primary site (89.0%, *n* = 967). In the analysis of cancer‐specific deaths, the composition of the patient population was dominated by males (55.7%, *n* = 548), unilateral in laterality (98.4%, *n* = 967), clinical stage IV (65.2%, *n* = 641), T4 (43.6%, *n* = 429), N2 (50.5%, *n* = 496), and M1 (65.2%, *n* = 641) predominated. Most patients did not undergo surgical treatment (84.3%, *n* = 829) and did not undergo surgery other than the primary focus (88.9%, *n* = 874) (Tables 1 and 2).

**TABLE 1:** Description of baseline characteristics of patients with PLCNEC.

Variable	Cases	Data before PSM	Data after PSM	*p*			*p*
		Radiotherapy = No	Radiotherapy = Yes		Radiotherapy = No	Radiotherapy = Yes	
	(*N* = 1086), *n* (%)	(*N* = 431), *n* (%)	(*N* = 655), *n* (%)		(*N* = 366), *n* (%)	(*N* = 366), *n* (%)	
Race				0.187			0.826
White	907 (83.5)	367 (85.2)	540 (82.4)		310 (84.7)	315 (86.1)	
Black	129 (11.9)	42 (9.7)	87 (13.3)		39 (10.7)	37 (10.1)	
Other	50 (4.6)	22 (5.1)	28 (4.3)		17 (4.6)	14 (3.8)	
Sex				0.332			1.000
Male	599 (55.2)	246 (57.1)	353 (53.9)		207 (56.6)	207 (56.6)	
Female	487 (44.8)	185 (42.9)	302 (46.1)		159 (43.4)	159 (43.4)	
Laterality				0.102			0.865
Left	439 (40.4)	190 (44.1)	249 (38.0)		156 (42.6)	164 (44.8)	
Right	630 (58.0)	235 (54.5)	395 (60.3)		207 (56.6)	198 (54.1)	
Bilateral	10 (0.9)	5 (1.2)	5 (0.8)		2 (0.5)	2 (0.5)	
Unknown	7 (0.6)	1 (0.2)	6 (0.9)		1 (0.3)	2 (0.5)	
Stage				0.012			0.817
III	406 (37.4)	141 (32.7)	265 (40.5)		129 (35.2)	133 (36.3)	
IV	680 (62.6)	290 (67.3)	390 (59.5)		237 (64.8)	233 (63.7)	
T stage				0.027			0.740
T1	157 (14.5)	53 (12.3)	104 (15.9)		47 (12.8)	38 (10.4)	
T2	360 (33.1)	143 (33.2)	217 (33.1)		128 (35.0)	125 (34.2)	
T3	66 (6.1)	23 (5.3)	43 (6.6)		19 (5.2)	19 (5.2)	
T4	468 (43.1)	190 (44.1)	278 (42.4)		164 (44.8)	172 (47.0)	
Tx	35 (3.2)	22 (5.1)	13 (2.0)		8 (2.2)	12 (3.3)	
N stage				0.687			0.872
N0	195 (18.0)	73 (16.9)	122 (18.6)		61 (16.7)	71 (19.4)	
N1	85 (7.8)	39 (9.0)	46 (7.0)		30 (8.2)	30 (8.2)	
N2	558 (51.4)	221 (51.3)	337 (51.5)		189 (51.6)	180 (49.2)	
N3	221 (20.3)	89 (20.6)	132 (20.2)		77 (21.0)	74 (20.2)	
Nx	27 (2.5)	9 (2.1)	18 (2.7)		9 (2.5)	11 (3.0)	
M stage				0.012			0.817
M0	406 (37.4)	141 (32.7)	265 (40.5)		129 (35.2)	133 (36.3)	
M1	680 (62.6)	290 (67.3)	390 (59.5)		237 (64.8)	233 (63.7)	
Age							
< 65	534 (49.2)	181 (42.0)	353 (53.9)	< 0.001	172 (47.0)	166 (45.4)	0.711
≥ 65	552 (50.8)	250 (58.0)	302 (46.1)		194 (53.0)	200 (54.6)	
Tumor size, cm				0.224			0.767
≤ 3	336 (30.9)	138 (32.0)	198 (30.2)		109 (29.8)	97 (26.5)	
(3,5]	321 (29.6)	136 (31.6)	185 (28.2)		115 (31.4)	120 (32.8)	
(5,7]	213 (19.6)	84 (19.5)	129 (19.7)		73 (19.9)	73 (19.9)	
> 7	216 (19.9)	73 (16.9)	143 (21.8)		69 (18.9)	76 (20.8)	
Marital				0.298			0.594
Single	162 (14.9)	71 (16.5)	91 (13.9)		64 (17.5)	51 (13.9)	
Married	609 (56.1)	244 (56.6)	365 (55.7)		202 (55.2)	214 (58.5)	
Other	282 (26.0)	107 (24.8)	175 (26.7)		91 (24.9)	93 (25.4)	
Unknown	33 (3.0)	9 (2.1)	24 (3.7)		9 (2.5)	8 (2.2)	
Primary site				0.020			0.930
Upper	243 (22.4)	112 (26.0)	131 (20.0)		89 (24.3)	81 (22.1)	
Middle	47 (4.3)	24 (5.6)	23 (3.5)		19 (5.2)	17 (4.6)	
Lower	643 (59.2)	232 (53.8)	411 (62.7)		210 (57.4)	213 (58.2)	
Main bronchus	61 (5.6)	30 (7.0)	31 (4.7)		22 (6.0)	22 (6.0)	
Overlapping lesion	14 (1.3)	6 (1.4)	8 (1.2)		5 (1.4)	6 (1.6)	
Lung, NOS	78 (7.2)	27 (6.3)	51 (7.8)		21 (5.7)	27 (7.4)	
Grade				0.401			0.985
I	8 (0.7)	4 (0.9)	4 (0.6)		3 (0.8)	2 (0.5)	
II	8 (0.7)	3 (0.7)	5 (0.8)		3 (0.8)	3 (0.8)	
III	339 (31.2)	141 (32.7)	198 (30.2)		116 (31.7)	121 (33.1)	
IV	111 (10.2)	51 (11.8)	60 (9.2)		37 (10.1)	35 (9.6)	
Unknown	620 (57.1)	232 (53.8)	388 (59.2)		207 (56.6)	205 (56.0)	
Surgery				0.027			1.000
No	897 (82.6)	342 (79.4)	555 (84.7)		300 (82.0)	299 (81.7)	
Yes	189 (17.4)	89 (20.6)	100 (15.3)		66 (18.0)	67 (18.3)	
Other surgery				< 0.001			0.641
No	967 (89.0)	405 (94.0)	562 (85.8)		342 (93.4)	346 (94.5)	
Yes	119 (11.0)	26 (6.0)	93 (14.2)		24 (6.6)	20 (5.5)	

Abbreviation: PLCNEC, pulmonary large cell neuroendocrine carcinoma.

**TABLE 2:** Description of baseline characteristics of cancer‐specific patients with PLCNEC.

Variable	Cases	Data before PSM			Data after PSM		
		Radiotherapy = No	Radiotherapy = Yes	*p*	Radiotherapy = No	Radiotherapy = Yes	*p*
	(*N* = 983), *n* (%)	(*N* = 394), *n* (%)	(*N* = 589), *n* (%)		(*N* = 316), *n* (%)	(*N* = 316), *n* (%)	
Race				0.299			0.705
White	821 (83.5)	335 (85.0)	486 (82.5)		270 (85.4)	266 (84.2)	
Black	116 (11.8)	39 (9.9)	77 (13.1)		32 (10.1)	38 (12.0)	
Other	46 (4.7)	20 (5.1)	26 (4.4)		14 (4.4)	12 (3.8)	
Sex				0.614			0.809
Male	548 (55.7)	224 (56.9)	324 (55.0)		180 (57.0)	184 (58.2)	
Female	435 (44.3)	170 (43.1)	265 (45.0)		136 (43.0)	132 (41.8)	
Laterality				0.166			0.853
Left	395 (40.2)	172 (43.7)	223 (37.9)		139 (44.0)	134 (42.4)	
Right	572 (58.2)	217 (55.1)	355 (60.3)		175 (55.4)	178 (56.3)	
Bilateral	9 (0.9)	4 (1.0)	5 (0.8)		1 (0.3)	2 (0.6)	
Unknown	7 (0.7)	1 (0.3)	6 (1.0)		1 (0.3)	2 (0.6)	
Stage				0.011			0.800
III	342 (34.8)	118 (29.9)	224 (38.0)		107 (33.9)	103 (32.6)	
IV	641 (65.2)	276 (70.1)	365 (62.0)		209 (66.1)	213 (67.4)	
T stage				0.033			0.722
T1	134 (13.6)	46 (11.7)	88 (14.9)		38 (12.0)	47 (14.9)	
T2	323 (32.9)	129 (32.7)	194 (32.9)		114 (36.1)	109 (34.5)	
T3	64 (6.5)	22 (5.6)	42 (7.1)		19 (6.0)	16 (5.1)	
T4	429 (43.6)	176 (44.7)	253 (43.0)		140 (44.3)	136 (43.0)	
Tx	33 (3.4)	21 (5.3)	12 (2.0)		5 (1.6)	8 (2.5)	
N stage				0.486			0.776
N0	173 (17.6)	66 (16.8)	107 (18.2)		50 (15.8)	62 (19.6)	
N1	82 (8.3)	39 (9.9)	43 (7.3)		31 (9.8)	28 (8.9)	
N2	496 (50.5)	197 (50.0)	299 (50.8)		162 (51.3)	154 (48.7)	
N3	208 (21.2)	85 (21.6)	123 (20.9)		69 (21.8)	67 (21.2)	
Nx	24 (2.4)	7 (1.8)	17 (2.9)		4 (1.3)	5 (1.6)	
M stage				0.011			0.800
M0	342 (34.8)	118 (29.9)	224 (38.0)		107 (33.9)	103 (32.6)	
M1	641 (65.2)	276 (70.1)	365 (62.0)		209 (66.1)	213 (67.4)	
Age				0.002			0.873
< 65	486 (49.4)	170 (43.1)	316 (53.7)		143 (45.3)	146 (46.2)	
≥ 65	497 (50.6)	224 (56.9)	273 (46.3)		173 (54.7)	170 (53.8)	
Tumor size, cm				0.150			0.935
≤ 3	293 (29.8)	125 (31.7)	168 (28.5)		88 (27.8)	94 (29.7)	
(3,5]	285 (29.0)	122 (31.0)	163 (27.7)		96 (30.4)	97 (30.7)	
(5,7]	200 (20.3)	78 (19.8)	122 (20.7)		69 (21.8)	64 (20.3)	
> 7	205 (20.9)	69 (17.5)	136 (23.1)		63 (19.9)	61 (19.3)	
Marital				0.479			0.962
Single	144 (14.6)	63 (16.0)	81 (13.8)		45 (14.2)	44 (13.9)	
Married	550 (56.0)	221 (56.1)	329 (55.9)		181 (57.3)	182 (57.6)	
Other	258 (26.2)	101 (25.6)	157 (26.7)		81 (25.6)	83 (26.3)	
Unknown	31 (3.2)	9 (2.3)	22 (3.7)		9 (2.8)	7 (2.2)	
Primary site				0.034			0.895
Upper	213 (21.7)	98 (24.9)	115 (19.5)		73 (23.1)	68 (21.5)	
Middle	44 (4.5)	23 (5.8)	21 (3.6)		18 (5.7)	18 (5.7)	
Lower	585 (59.5)	215 (54.6)	370 (62.8)		188 (59.5)	184 (58.2)	
Main bronchus	56 (5.7)	28 (7.1)	28 (4.8)		18 (5.7)	19 (6.0)	
Overlapping lesion	14 (1.4)	6 (1.5)	8 (1.4)		4 (1.3)	6 (1.9)	
Lung, NOS	71 (7.2)	24 (6.1)	47 (8.0)		15 (4.7)	21 (6.6)	
Grade				0.521			0.866
I	8 (0.8)	4 (1.0)	4 (0.7)		1 (0.3)	2 (0.6)	
II	6 (0.6)	2 (0.5)	4 (0.7)		2 (0.6)	1 (0.3)	
III	302 (30.7)	121 (30.7)	181 (30.7)		105 (33.2)	97 (30.7)	
IV	101 (10.3)	48 (12.2)	53 (9.0)		31 (9.8)	35 (11.1)	
Unknown	566 (57.6)	219 (55.6)	347 (58.9)		177 (56.0)	181 (57.3)	
Surgery				0.301			0.915
No	829 (84.3)	326 (82.7)	503 (85.4)		265 (83.9)	263 (83.2)	
Yes	154 (15.7)	68 (17.3)	86 (14.6)		51 (16.1)	53 (16.8)	
Other surgery				< 0.001			1.000
No	874 (88.9)	369 (93.7)	505 (85.7)		292 (92.4)	291 (92.1)	
Yes	109 (11.1)	25 (6.3)	84 (14.3)		24 (7.6)	25 (7.9)	

Abbreviation: PLCNEC, pulmonary large cell neuroendocrine carcinoma.

### 3.2 PSM and KM Analysis

Following 1:1 PSM matching, the clinical characteristics of the patients were balanced.

We found statistically significant differences in OS and CSS between the radiotherapy and chemotherapy‐only groups before and after PSM. Survival curves demonstrated superior survival outcomes in the combined radiotherapy group compared to the chemotherapy‐only group (Figure 2B,C,E,F). Whether before or after PSM, patients treated with radiotherapy had better OS and CSS than those treated with chemotherapy‐only. Before PSM, median OS was 11.0 months (95% CI, 11.0–12.0) in the chemoradiotherapy group and 10.0 months (95% CI, 9.0–11.0) in the chemotherapy‐only group (*p* = 0.009). Median CSS was 11.0 months (95% CI, 10.0–12.0) versus 9.0 months (95% CI, 9.0–11.0), respectively (*p* = 0.005). After PSM, the survival benefit remained statistically significant, with median OS of 11.0 (95% CI, 10.0–12.0) versus 10.0 (95% CI, 9.0–11.0) months (*p* = 0.049) and median CSS of 11.0 (95% CI, 9.0–12.0) versus 9.0 (95% CI, 8.0–11.0) months (*p* = 0.048).

After analyzing patients with clinical stages III and IV respectively in the same way, it was found that the chemoradiotherapy group and chemotherapy‐only group of patients in stage III had no statistically significant OS (*p* = 0.086) and statistically significant CSS (*p* = 0.029) before PSM; and both OS and CSS were statistically significant (*p* = 0.008, *p* = 0.008) after PSM. The results showed that patients who received chemoradiotherapy at stage III had better OS and CSS (5‐year survival 93.9% vs. 84.8%, *p* = 0.008; 5‐year CSS rate 90.8% vs. 83.9%, *p* = 0.008), and patients at stage IV had no statistically significant difference between the chemoradiotherapy and chemotherapy‐only groups in terms of both OS and CSS before and after PSM (pre‐PSM: *p* = 0.38, *p* = 0.503; post PSM: *p* = 0.631, *p* = 0.21).

### 3.3 Multicollinearity

Multicollinearity among covariates was assessed using generalized variance inflation factors (GVIFs). All variables exhibited GVIF values < 5 and adjusted GVIF values < 2, indicating negligible multicollinearity (Table 3).

**TABLE 3:** Multi collinearity test in multivariate analysis.

Variable	GVIF	Df	Adjusted GVIF
Sex	1.004078	1	1.002037
N stage	1.117393	4	1.013972
M stage	1.196203	1	1.093711
Surgery	1.194825	1	1.093081
Radiotherapy	1.020319	1	1.010109

Abbreviations: Adjusted GVIF, GVIF^(1/(2*Df)); Df, degree of freedom; GVIF, generalized variance inflation factor.

### 3.4 Survival Analyses of Cox Regression

We employed univariate and multivariate Cox regression analyses on the training cohort to ascertain the prognostic factors for OS and CSS in patients with stage III–IV PLCNEC treated with chemotherapy. Our analysis revealed that sex, clinical N stage, clinical M stage, surgery, and radiotherapy were independent prognostic factors for OS and CSS (*p* < 0.05) (Tables 4 and 5).

**TABLE 4:** Univariable and multivariable Cox regression model analysis of OS.

Variable	Univariate			Multivariate		
	HR	95% CI	*p*	HR	95% CI	*p*
Sex						
Male	—	—		—	—	
Female	0.73	0.63, 0.84	< 0.001	0.71	0.61, 0.82	< 0.001
Stage						
III	—	—				
IV	2.19	1.87, 2.56	< 0.001			
M stage						
M0	—	—		—	—	
M1	2.19	1.87, 2.56	< 0.001	1.81	1.53, 2.15	< 0.001
Age						
65	—	—		—	—	
≥ 65	1.19	1.03, 1.38	0.018	1.12	0.96, 1.30	0.140
Surgery						
No	—	—		—	—	
Yes	0.40	0.32, 0.49	< 0.001	0.49	0.39, 0.63	< 0.001
Other surgery						
No	—	—				
Yes	1.01	0.80, 1.28	0.920			
Radiotherapy						
No	—	—		—	—	
Yes	0.78	0.67, 0.91	0.001	0.73	0.63, 0.86	< 0.001
Race						
White	—	—				
Black	1.03	0.83, 1.29	0.769			
Other	1.0	0.70, 1.41	0.975			
Laterality						
Left	—	—				
Right	1.14	0.98, 1.32	0.092			
Bilateral	1.69	0.84, 3.42	0.143			
Unknown	2.30	1.02, 5.17	0.045			
T stage						
T1	—	—		—	—	
T2	1.01	0.80, 1.28	0.917	1.00	0.79, 1.27	0.973
T3	0.93	0.65, 1.31	0.667	1.02	0.71, 1.46	0.915
T4	1.27	1.02, 1.59	0.031	1.12	0.90, 1.41	0.307
Tx	1.33	0.85, 2.09	0.213	0.84	0.53, 1.34	0.473
N stage						
N0	—	—		—	—	
N1	0.83	0.59, 1.15	0.258	0.84	0.60, 1.18	0.312
N2	0.93	0.77, 1.13	0.461	1.08	0.88, 1.31	0.455
N3	1.57	1.25, 1.98	< 0.001	1.48	1.17, 1.87	0.001
Nx	1.74	1.05, 2.89	0.031	1.81	1.08, 3.04	0.024
Tumor size, cm						
≤ 3	—	—				
(3, 5]	1.09	0.91, 1.31	0.364			
(5, 7]	1.09	0.88, 1.34	0.437			
7	1.23	1.00, 1.52	0.053			
Marital						
Single	—	—				
Married	0.90	0.73, 1.12	0.350			
Other	0.90	0.71, 1.14	0.380			
Unknown	0.80	0.49, 1.31	0.380			
Primary site						
Upper	—	—				
Middle	1.27	0.88, 1.84	0.196			
Lower	0.96	0.80, 1.15	0.660			
Main bronchus	1.40	1.00, 1.96	0.051			
Overlapping lesion	0.97	0.51, 1.84	0.922			
Lung, NOS	1.17	0.86, 1.59	0.311			
Grade						
I	—	—				
II	0.80	0.25, 2.53	0.709			
III	0.98	0.46, 2.08	0.961			
IV	1.28	0.59, 2.79	0.526			
Unknown	1.34	0.63, 2.82	0.448			

Abbreviations: CI, confidence interval; HR, hazard ratio; OS, overall survival.

**TABLE 5:** Univariable and multivariable Cox regression model analysis of CSS.

Characteristic	Univariate			Multivariate		
	HR	95% CI	*p*	HR	95% CI	*p*
Sex						
Male	—	—		—	—	
Female	0.76	0.65, 0.89	< 0.001	0.74	0.63, 0.86	< 0.001
Stage						
III	—	—				
IV	1.99	1.69, 2.35	< 0.001			
M stage						
M0	—	—		—	—	
M1	1.99	1.69, 2.35	< 0.001	1.78	1.48, 2.14	< 0.001
Age						
65	—	—		—	—	
≥ 65	1.19	1.02, 1.39	0.025	1.17	1.0, 1.37	0.058
Surgery						
No	—	—		—	—	
Yes	0.39	0.31, 0.49	< 0.001	0.53	0.42, 0.69	< 0.001
Other surgery						
No	—	—				
Yes	0.83	0.64, 1.06	0.139			
Radiotherapy						
No	—	—		—	—	
Yes	0.75	0.64, 0.87	< 0.001	0.77	0.65, 0.90	0.002
Race						
White	—	—				
Black	0.98	0.77, 1.24	0.841			
Other	1.29	0.90, 1.84	0.164			
Laterality						
Left	—	—		—	—	
Right	1.16	0.99, 1.36	0.061	1.15	0.98, 1.36	0.095
Bilateral	2.94	1.30, 6.63	0.009	2.13	0.88, 5.12	0.092
Unknown	2.32	0.96, 5.64	0.062	2.14	0.80, 5.75	0.132
T stage						
T1	—	—		—	—	
T2	0.98	0.76, 1.26	0.856	0.97	0.75, 1.26	0.824
T3	0.85	0.58, 1.24	0.404	0.96	0.64, 1.42	0.830
T4	1.32	1.03, 1.68	0.028	1.16	0.90, 1.49	0.249
Tx	1.37	0.85, 2.19	0.192	0.91	0.55, 1.51	0.723
N stage						
N0	—	—		—	—	
N1	0.72	0.52, 1.00	0.052	0.87	0.62, 1.23	0.443
N2	0.97	0.78, 1.20	0.787	1.17	0.93, 1.46	0.178
N3	1.57	1.22, 2.02	< 0.001	1.48	1.13, 1.92	0.004
Nx	1.46	0.88, 2.44	0.146	1.33	0.77, 2.30	0.309
Tumor size, cm						
≤ 3	—	—				
(3, 5]	1.05	0.86, 1.28	0.652			
(5, 7]	1.19	0.95, 1.49	0.123			
7	1.22	0.98, 1.52	0.073			
Marital						
Single	—	—				
Married	1.00	0.80, 1.24	0.985			
Other	0.94	0.73, 1.21	0.640			
Unknown	1.09	0.69, 1.72	0.725			
Primary site						
Upper	—	—		—	—	
Middle	1.13	0.76, 1.69	0.550	1.02	0.67, 1.54	0.931
Lower	1.05	0.86, 1.27	0.629	1.02	0.83, 1.24	0.880
Main bronchus	1.58	1.11, 2.26	0.011	1.17	0.81, 1.68	0.410
Overlapping lesion	0.87	0.44, 1.71	0.687	0.89	0.45, 1.77	0.741
Lung, NOS	1.23	0.89, 1.70	0.214	0.97	0.67, 1.39	0.855
Grade						
I	—	—				
II	1.45	0.39, 5.40	0.581			
III	0.88	0.36, 2.14	0.778			
IV	1.02	0.41, 2.54	0.963			
Unknown	1.17	0.49, 2.84	0.722			

Abbreviations: CI, confidence interval; CSS, cancer‐specific survival; HR, hazard ratio.

### 3.5 Nomogram for OS and CSS

**FIGURE 3:**
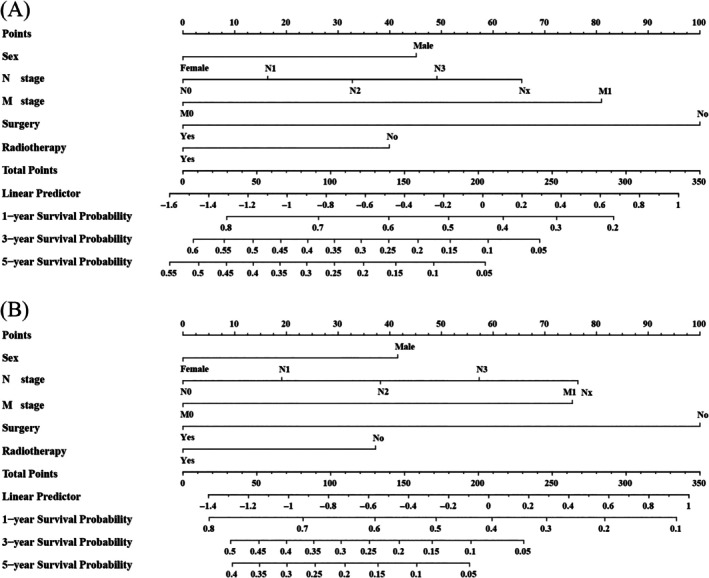
(A) The nomogram of predicting OS in PLCNEC patients undergoing chemotherapy in stages III–IV; (B) The nomogram of predicting CSS in PLCNEC patients undergoing chemotherapy in stages III–IV. CSS, cancer‐specific survival; OS, overall survival; PLCNEC, pulmonary large cell neuroendocrine carcinoma.

**FIGURE 4:**
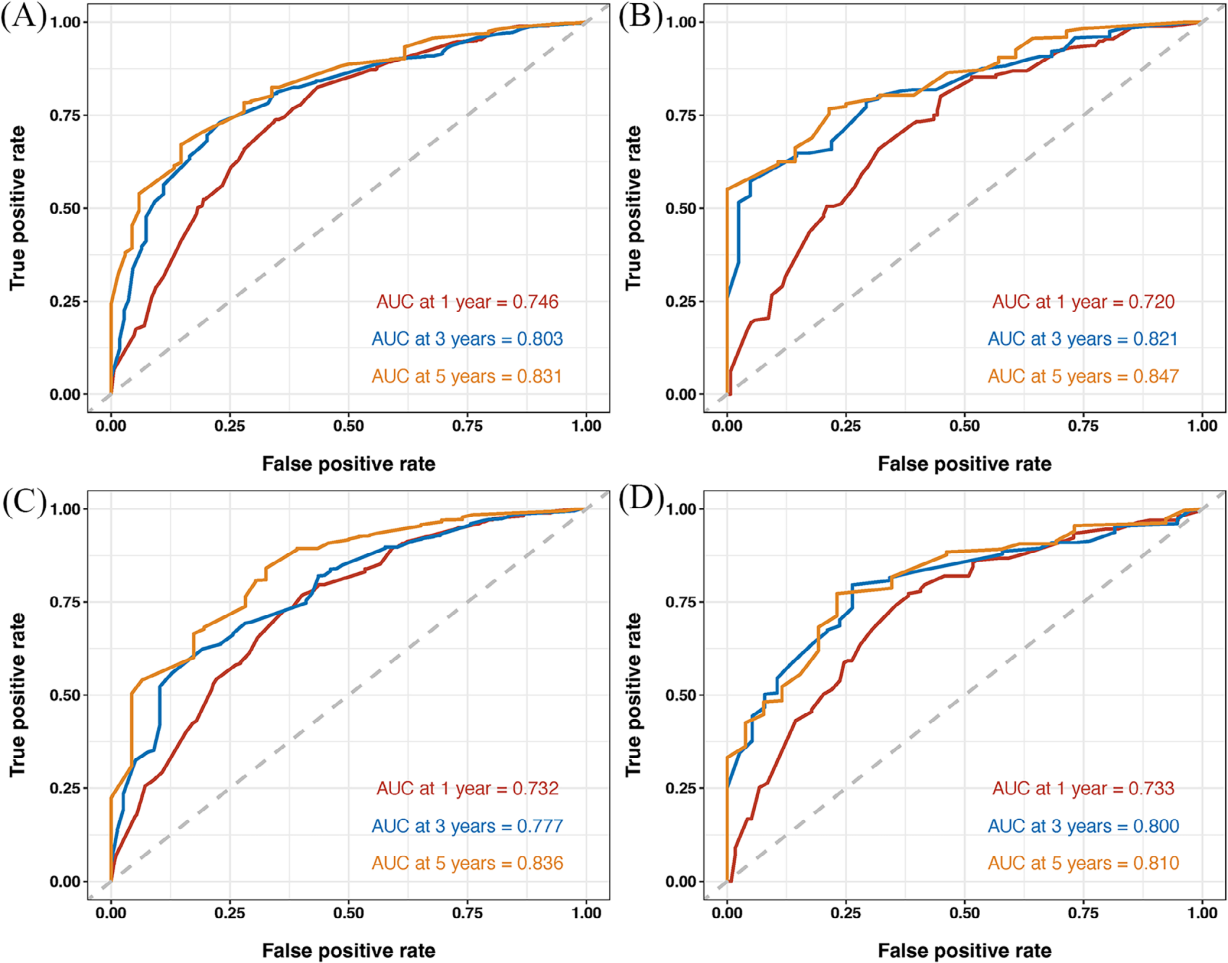
(A) The ROC curves of the training cohort (OS); (B) The ROC curves of the validation cohort (OS); (C) The ROC curves of the training cohort (CSS); (D) The ROC curves of the validation cohort (CSS). AUC, area under curve; CSS, cancer‐specific survival; OS, overall survival; ROC, receiver operating characteristic.

**FIGURE 5:**
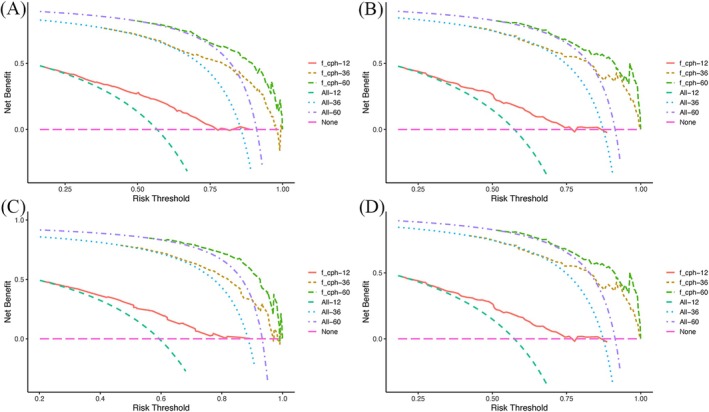
(A, B, C) The 1‐year, 3‐year, and 5‐year calibration plots of the training cohort (OS); (D, E, F) The 1‐year, 3‐year, and 5‐year calibration plots of the validation cohort (OS). OS, overall survival.

**FIGURE 6:**
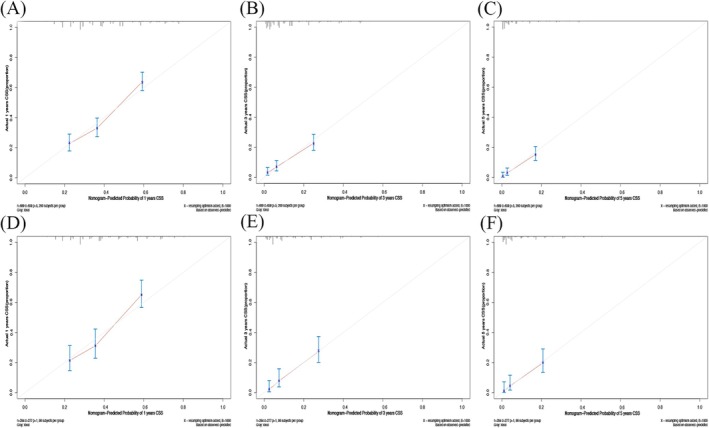
(A, B, C) The 1‐year, 3‐year, and 5‐year calibration plots of the training cohort (CSS); (D, E, F) The 1‐year, 3‐year, and 5‐year calibration plots of the validation cohort (CSS). CSS, cancer‐specific survival.

**FIGURE 7:**
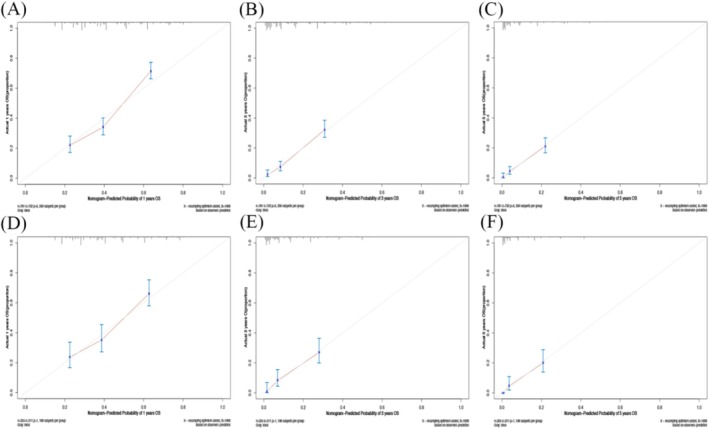
(A) The DCA curves of the training cohort (OS); (B) The DCA curves of the validation cohort (OS); (C) The DCA curves of the training cohort (CSS); (D) The DCA curves of the validation cohort (CSS). CSS, cancer‐specific survival; DCA, decision curve analysis; OS, overall survival.

Nomograms were developed to predict OS and CSS of patients with PLCNEC (Figure 3). These nomograms were constructed using a set of five independent risk factors, including sex, clinical N stage, clinical M stage, surgery, and radiotherapy. The discriminative performance of the nomograms was evaluated using receiver operating characteristic (ROC) curves. In internal validation, the models demonstrated good predictive ability, with areas under the curve (AUCs) for OS and CSS (Figure 4). The calibration curves indicate good agreement between predicted and observed survival probabilities in both the training and validation cohorts (Figures 5 and 6). The decision curve analysis further demonstrated that the nomograms provided a favorable net clinical benefit across a range of threshold probabilities in both cohorts, supporting their potential utility for individualized survival prediction (Figure 7).

### 3.6 Clinical Application of the Nomograms

To illustrate the clinical applicability of the nomograms, a representative application scenario is presented based on Figure 3. For example, a male patient with stage III PLCNEC receiving chemotherapy, presenting with N2 disease, M0 status, no surgical resection, and planned radiotherapy would be assigned points for each variable according to the nomogram.

In the OS nomogram (Figure 3A), this patient would receive approximately 45 points for male sex, 33 points for N2 stage, 0 points for M0 stage, 100 points for no surgery, and 0 points for receipt of radiotherapy, yielding a total score of approximately 178 points (Figure S1). By projecting this total score onto the survival probability scales, the estimated 1‐, 3‐, and 5‐year OS probabilities are approximately 51%, 20%, and 9%, respectively. Using the CSS nomogram (Figure 3B), a similar scoring process applies. These examples demonstrate how the nomograms can be applied in clinical practice to generate individualized survival estimates and support prognostic assessment and treatment decision‐making.

### 3.7 Subgroup Analysis

**FIGURE 8:**
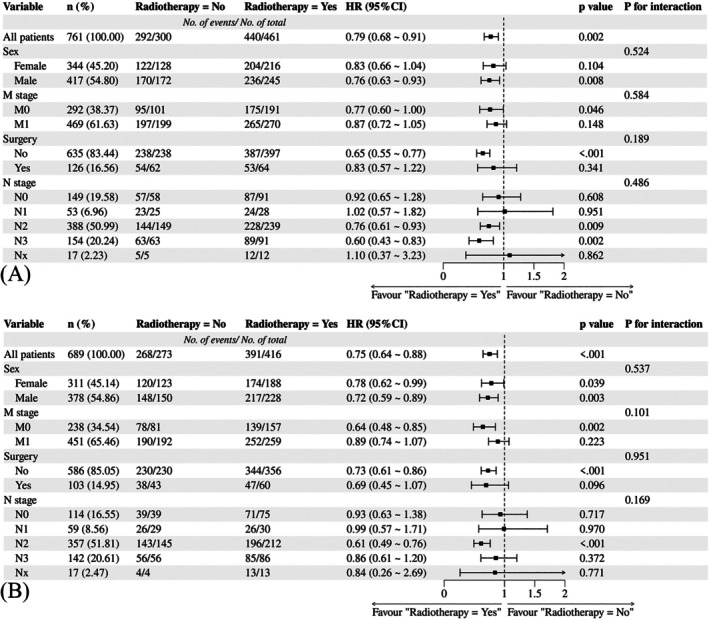
(A) The forest plot and subgroup analyses of OS; (B) The forest plot and subgroup analyses of CSS. CSS, cancer‐specific survival; OS, overall survival.

In order to explore differences in the effects of radiotherapy in different subgroups of the population and potential interactions, subgroup analyses were performed (Figure 8). The results demonstrated that radiotherapy exhibited a substantial improvement in OS (HR = 0.79, *p* = 0.002) and CSS (HR = 0.75, *p* < 0.001). When analyzing OS, it was observed that men (HR = 0.76), non‐operated patients (HR = 0.65), N2 (HR = 0.76), and N3 (HR = 0.60) patients could significantly benefit from radiotherapy. However, no significant interaction effect on OS was identified. In the analysis of CSS, the following groups demonstrated significant benefits from radiotherapy: females (HR = 0.78, *p* = 0.039), males (HR = 0.72, *p* = 0.003), M0 (HR = 0.64, p = 0.002), non‐operated patients (HR = 0.73, *p* < 0.001), and N2 (HR = 0.61, p < 0.001). However, no significant interaction effect was observed on CSS as well.

## 4 Discussion

Pulmonary large cell neuroendocrine carcinoma (PLCNEC) is an uncommon but highly aggressive tumor, accounting for only 2%–3% of all lung cancers; however, given the high global incidence of lung cancer, PLCNEC still represents a clinically significant population [14]. Consistent with previous reports, a substantial proportion of patients in our study presented with stage III–IV disease, reflecting the rapid progression and poor biological behavior of PLCNEC [7, 15]. While surgery remains the optimal treatment option for resectable stage I–IIIA cases [16], its effectiveness diminishes in advanced disease, and chemotherapy becomes the mainstay of treatment [17, 18, 19]. Nevertheless, the role of radiotherapy in stage III–IV PLCNEC receiving chemotherapy has remained ambiguous, prompting the need for more refined prognostic assessment and treatment guidance.

Accumulating evidence suggests that multiple clinicopathological and treatment‐related factors, including sex, nodal status, metastatic status, surgical intervention, and radiotherapy, may jointly influence prognosis in patients with advanced PLCNEC. These associations are broadly consistent with prior studies indicating that both tumor burden and treatment intensity play important roles in determining outcomes in this aggressive malignancy [20]. The differential impact of radiotherapy across disease stages suggests that its clinical benefit is highly context‐dependent, particularly with respect to disease extent and treatment intent. Previous studies in LCNEC and SCLC populations similarly indicate that radiotherapy is more likely to translate into a survival benefit in locoregionally confined disease than in widely metastatic settings [21].

From a clinical perspective, the observed survival benefit in stage III PLCNEC likely reflects the role of radiotherapy delivered with radical or definitive intent, aiming to enhance locoregional tumor control in patients with relatively limited disease burden. Similar to locally advanced small cell lung cancer and selected LCNEC cases, aggressive local radiotherapy combined with systemic chemotherapy may reduce locoregional failure and delay disease progression, thereby translating into improved survival outcomes.

In contrast, radiotherapy in stage IV PLCNEC is more commonly administered with consolidative or palliative intent rather than curative intent. The absence of a significant population‐level survival benefit in stage IV disease may be attributed to higher systemic tumor burden, early dissemination, and the predominance of distant progression, which collectively diminish the relative contribution of local control to overall survival.

Importantly, the absence of a population‐level survival benefit does not imply that radiotherapy is universally ineffective in stage IV disease. From a clinical standpoint, stage IV PLCNEC should not be viewed as a homogeneous “non‐beneficial” population with respect to radiotherapy. Rather, it represents a biologically and clinically heterogeneous group requiring individualized treatment strategies. In selected patients with oligometastatic disease or dominant thoracic lesions, local radiotherapy delivered with consolidative intent may contribute to improved disease control, as suggested by evidence from LCNEC, SCLC, and other thoracic malignancies [22, 23, 24].

Moreover, beyond traditional survival endpoints such as OS and CSS, radiotherapy plays a critical role in non‐survival outcomes for stage IV patients, including local tumor control, symptom palliation, and quality‐of‐life improvement. Palliative thoracic radiotherapy has been shown to effectively relieve symptoms such as dyspnea, hemoptysis, and chest pain and remains an essential component of multidisciplinary management in advanced lung cancer [25].

Taken together, these considerations support an individualized approach to radiotherapy decision‐making in stage IV PLCNEC, where patient selection should be guided by metastatic burden, disease distribution, symptom severity, and overall treatment goals, rather than survival outcomes alone. Importantly, the findings of this study should be interpreted as associations rather than causal effects. Although propensity score matching was applied to reduce baseline imbalances, residual confounding due to unmeasured variables—such as performance status, comorbidities, and treatment intent—cannot be excluded. Therefore, the observed survival differences may partly reflect treatment selection bias rather than a purely causal effect of radiotherapy.

Beyond disease stage and treatment intent, emerging biological evidence may help explain the differential prognostic impact of radiotherapy. In recent years, accumulating molecular and translational studies have demonstrated that PLCNEC is not a biologically uniform entity, but rather comprises at least two major molecular subtypes with distinct genomic profiles, treatment sensitivities, and prognostic implications. One subgroup, commonly referred to as the “SCLC‐like” subtype, is characterized by concurrent inactivation of TP53 and RB1, high proliferative activity, and transcriptional features resembling small cell lung cancer. This subtype has been reported to exhibit greater sensitivity to platinum‐based chemotherapy and radiotherapy, consistent with its aggressive yet radiosensitive biological behavior [3, 26, 27]. In contrast, the “NSCLC‐like” subtype typically lacks RB1 inactivation and harbors genomic alterations more frequently observed in non‐small cell lung cancer, such as KRAS, STK11, or KEAP1 mutations. Emerging evidence suggests that these tumors may demonstrate relative resistance to conventional platinum‐based regimens and potentially different responses to radiotherapy, contributing to heterogeneity in clinical outcomes [13, 14, 26]. More recently, large‐scale genomic and transcriptomic analyses have further refined this molecular classification, revealing that SCLC‐like and NSCLC‐like PLCNEC subtypes also differ in immune microenvironment features, DNA damage–repair pathways, and transcriptional programs, which may influence sensitivity to both systemic therapy and radiotherapy [14, 28]. In addition, tumor microenvironmental factors (e.g., hypoxic niches) and alterations in DNA damage–repair capacity may further modulate radiation response in PLCNEC [29]. These findings reinforce the concept that molecular heterogeneity is a key determinant of treatment response in high‐grade pulmonary neuroendocrine carcinomas.

Although molecular subtype information is unavailable in the SEER database, this emerging classification provides a plausible biological explanatory framework for the heterogeneous survival benefits observed with chemotherapy and radiotherapy in our study. It is conceivable that the survival advantage associated with radiotherapy in stage III PLCNEC may be driven, at least in part, by a higher proportion of radiosensitive SCLC‐like tumors in locoregionally confined disease, whereas advanced‐stage disease likely encompasses a broader and more heterogeneous molecular spectrum. Future prospective studies integrating molecular profiling with treatment selection are therefore warranted to validate these hypotheses and to optimize personalized therapeutic strategies for PLCNEC.

From a clinical perspective, translating these biological and clinical insights into individualized prognostic assessment remains essential. The proposed nomograms integrate multiple prognostic dimensions beyond anatomical staging, thereby facilitating refined risk stratification and long‐term prognostic counseling in advanced PLCNEC. This observation regarding the nomogram's stronger long‐term predictive performance may be partly explained by the reduced influence of transient clinical fluctuations over longer time horizons, which allows the model to capture more stable prognostic signals and diminish noise inherent in short‐term data. For clinicians, this suggests that the nomogram may be particularly valuable for long‐term prognostic counseling and treatment planning. Nevertheless, given that the models were derived from retrospective SEER data from the pre‐immunotherapy era and lack molecular‐level variables, their applicability in contemporary treatment settings should be interpreted with appropriate caution.

While traditional TNM staging remains fundamental for anatomical disease classification, it does not fully capture the prognostic heterogeneity among patients within the same stage. In contrast, the nomogram integrates multiple prognostic dimensions, including patient characteristics and treatment‐related factors such as surgery and radiotherapy. As a result, it provides complementary prognostic information beyond TNM staging and enables more refined individualized risk stratification within the same TNM stage.

With the rapid evolution of lung cancer treatments, it is also important to recognize that our study period (2004–2015) reflects the pre‐immunotherapy era. Although the present analysis was conducted in the pre‐immunotherapy era, accumulating biological and translational evidence suggests that PLCNEC may harbor features associated with potential responsiveness to immunotherapy. High‐grade pulmonary neuroendocrine carcinomas, including PLCNEC, are characterized by high proliferative activity, genomic instability, and, in a subset of cases, elevated tumor mutational burden, which may facilitate neoantigen generation and immune recognition [9, 10, 18].

In addition, increasing evidence supports a synergistic interaction between radiotherapy and immunotherapy, whereby radiotherapy can induce immunogenic cell death, enhance tumor antigen release and presentation, upregulate immune checkpoint pathways, and modulate the tumor microenvironment, thereby promoting systemic antitumor immune responses beyond the irradiated field. This biological rationale, often referred to as the “abscopal effect,” has been reported across multiple thoracic malignancies and provides a theoretical basis for combining radiotherapy with immunotherapy in PLCNEC.

Although immunotherapy exposure and immune biomarkers are unavailable in the SEER database, these considerations suggest that radiotherapy may function not only as a local treatment modality but also as a potential immunomodulatory partner in the contemporary management of PLCNEC. Future prospective studies integrating immunotherapy, radiotherapy, and molecular profiling are warranted to clarify optimal treatment strategies and to update prognostic models for the immunotherapy era.

Despite the strengths of our analysis, several limitations must be acknowledged. First, as a retrospective study, selection bias and unmeasured confounding are unavoidable. Second, SEER lacks key clinical details such as radiotherapy dose, target volume, fractionation regimen, chemotherapy protocol, comorbidities, and performance status, which may influence survival outcomes and limit interpretation. Third, although our variable selection was based on statistical significance from univariate Cox analyses, we used a clinical–statistical combined approach rather than automated selection approaches such as LASSO regression or conducting robustness assessments such as bootstrap or cross‐validation, which may affect model stability. Finally, only internal validation was performed; external validation using independent multicenter or international cohorts is needed to confirm the generalizability of the nomogram.

In future work, we plan to collaborate with multiple centers and incorporate external datasets to validate the model across diverse populations. Such efforts may also facilitate deeper exploration of molecular features, radiotherapy response patterns, and optimal integration of systemic therapies, ultimately refining prognostic tools and treatment strategies for PLCNEC.

## 5 Conclusions

In conclusion, we investigated the survival outcomes and factors influencing the prognosis of stage III–IV PLCNEC treated with chemotherapy and constructed a prognostic nomogram for clinicians to predict survival outcomes. Our findings suggest that radiotherapy is associated with improved survival outcomes in stage III disease, whereas its role in stage IV PLCNEC appears more heterogeneous and context‐dependent. We also identified patient subgroups that may derive differential benefit from radiotherapy across disease stages, highlighting the importance of individualized radiotherapy decision‐making in advanced PLCNEC.

## Author Contributions

**Haitao Li:** data curation, investigation, methodology, resources. **Ting Lin:** conceptualization, methodology, writing – review and editing. **Zefeng Xie:** conceptualization, methodology, project administration, supervision, writing – review and editing. **Guantong Liu:** data curation, investigation, validation. **Yuze Zhao:** conceptualization, data curation, formal analysis, investigation, software, visualization, writing – original draft, writing – review and editing. **Yu Li:** data curation, investigation, methodology, resources, writing – review and editing.

## Funding

The authors have nothing to report.

## Ethics Statement

Ethical approval was not required for the study involving humans in accordance with the local legislation and institutional requirements. Written informed consent to participate in this study was not required from the participants or the participants' legal guardians/next of kin in accordance with the national legislation and the institutional requirements. No potentially identifiable images or data are presented in this study.

## Conflicts of Interest

The authors declare no conflicts of interest.

## Supporting information


**Figure S1:** Clinical application of the prognostic nomograms in PLCNEC. PLCNEC, pulmonary large cell neuroendocrine carcinoma.

## Data Availability

Publicly available datasets were analyzed in this study. This data can be found here: https://seer.cancer.gov/data/.
