# Supplementary material for: Patients With Stage III PLCNEC Advocated for Radiotherapy Combined With Chemotherapy While With Stage IV Focus on Individualized Management: Evidence From SEER
Source: Cancer Rep (Hoboken). 2026 Jun 3;9(6):e70573. doi: 10.1002/cnr2.70573 (PMC13239243; doi:10.1002/cnr2.70573)
Supplement: Supplementary file 1 — Figure S1: Clinical application of the prognostic nomograms in PLCNEC. PLCNEC, pulmonary large cell neuroendocrine carcinoma. [file CNR2-9-e70573-s001.docx]

**Patients with stage III PLCNEC advocated for radiotherapy combined with chemotherapy while with stage IV focus on individualized management: Evidence From SEER**


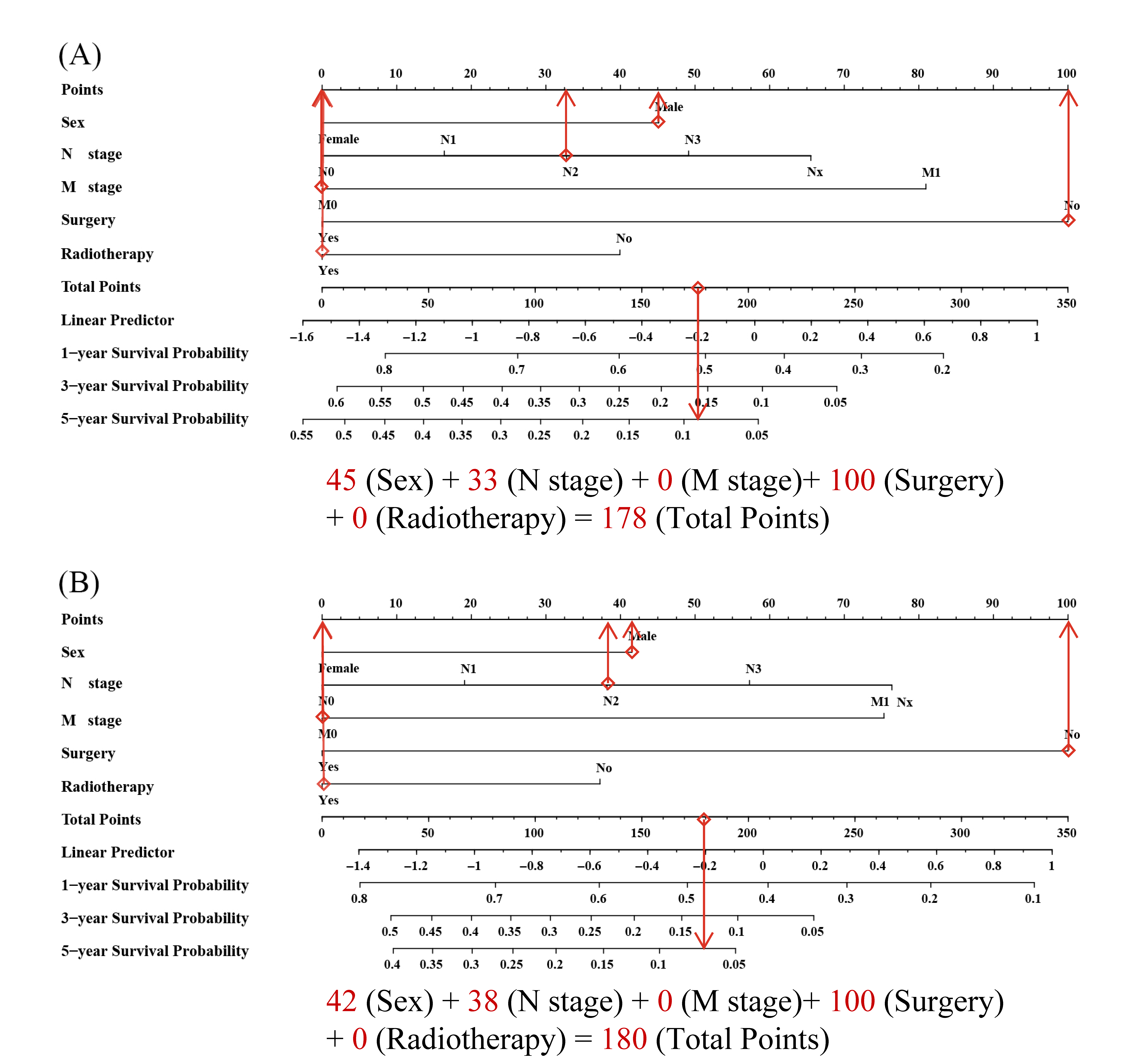


**FIGURE S1**. Clinical application of the prognostic nomograms in PLCNEC. PLCNEC, pulmonary large cell neuroendocrine carcinoma
